# sRACIPE 2.0: a systems biology circuit modeling toolkit for random circuit perturbation

**DOI:** 10.1093/bioinformatics/btag019

**Published:** 2026-01-19

**Authors:** Aidan Tillman, Daniel Ramirez, Mingyang Lu

**Affiliations:** Department of Bioengineering, Northeastern University, Boston, MA 02115, United States; Center for Theoretical Biological Physics, Northeastern University, Boston, MA 02115, United States; Department of Bioengineering, Northeastern University, Boston, MA 02115, United States; Center for Theoretical Biological Physics, Northeastern University, Boston, MA 02115, United States; Department of Bioengineering, Northeastern University, Boston, MA 02115, United States; Center for Theoretical Biological Physics, Northeastern University, Boston, MA 02115, United States

## Abstract

**Summary:**

The Random Circuit Perturbation (RACIPE) algorithm enables the exploration of the dynamical behaviors of gene regulatory circuits (GRCs) by simulating an ensemble of differential equation models via randomization of kinetic parameters. Here, we release sRACIPE 2.0, a major update to the R/Bioconductor package, as a unified platform for modeling GRCs with diverse interaction types using both deterministic and stochastic simulations. The update also introduces new features for modeling perturbation, extrinsic signaling and time-corrected noise, and a new diagnostic tool to ensure proper simulations. We hope that this release will serve as a versatile modeling toolkit for the systems biology community.

**Availability and implementation:**

The package is available on GitHub at https://github.com/lusystemsbio/sRACIPE under the MIT license. It is also available on Bioconductor at https://www.bioconductor.org/packages/release/bioc/html/sRACIPE.html.

## 1 Introduction

Gene Regulatory Circuits (GRCs) are systems of interacting genes that play crucial roles in cellular decision-making. GRCs have been studied for their influence on many cellular processes such as the Epithelial-Mesenchymal transition in cancer (Jia *et al.* 2019), the cell cycle (Katebi *et al.* 2020), the formation of lactose in *Escherichia coli* (Jacob and Monod 1961), and the circadian rhythm (Cox and Takahashi 2019). Typical approaches to study GRC dynamical behaviors include Boolean network models (Thakar 2024) and ordinary differential equation (ODE) models, though other approaches involving additional inputs such as differentiation trees or reference datasets have also been proposed (Karamveer and Uzun 2024). The Boolean approaches lack a quantitative resolution of the circuit’s behavior, as gene expression is discretized. Meanwhile, ODE models are limited by the presence of a large number of kinetic parameters, which can be difficult to determine from experiments and can heavily impact the model’s behavior. Furthermore, specific parametrization can fail to account for extrinsic noise to the system or cell-to-cell variability (Snijder and Pelkmans 2011). To address these issues, the algorithm of Random Circuit Perturbation (RACIPE) (Huang *et al.* 2017) was devised that models the dynamical behavior of a GRC using an ensemble of ODE models with randomly sampled kinetic parameters (detailed mathematical model in the Supplementary Information). To ensure that any given regulatory link is not over- or under-functional within the entire ensemble of models, RACIPE uses a “half-functional rule” to appropriately estimate the parameter ranges. RACIPE then simulates every model in the ensemble from one or more initial conditions to compute the steady-state gene expression of the GRC. Previous studies have shown that steady states from an ensemble of GRC models usually form distinct gene expression clusters, which can be associated with biological cellular states (Huang *et al.* 2017, Katebi *et al.* 2020). Moreover, RACIPE has been applied to optimize bioinformatics-generated GRCs by direct comparison of simulated and experimental gene expression data (Ramirez *et al.* 2020, Su *et al.* 2022, Katebi *et al.* 2024); it has also been systemically applied to all four-node circuits to identify GRC motifs according to certain GRC behavior, such as the spatial structure of states (Clauss and Lu 2023).

So far, we have developed multiple software packages that implement the RACIPE algorithm. First, a C package of RACIPE was developed for modeling transcriptional regulation (Huang *et al.* 2018). Second, sRACIPE 1.0 was developed as a R/Bioconductor package for stochastic analysis (Kohar and Lu 2018). Third, a Python package was devised to study oscillatory dynamics of a GRC, which had no stochastic simulation options but had greater tools for deterministic simulation for GRCs with more interaction types and automated limit cycle detection (Katebi *et al.* 2020). However, none of these implementations contains a complete suite for RACIPE tools, and it is cumbersome to switch between different packages to use different features. To address these issues and improve its functionality, we introduce sRACIPE 2.0, a major update of the R/Bioconductor sRACIPE package that integrates the tools of these previous implementations of RACIPE under a unified framework. We also introduce new simulation tools, such as methods for modeling extrinsic signaling and time-correlated noise. Lastly, we also devise new diagnostic tools for deterministic simulations to assist users in running statistically sound and computationally efficient simulations. Together, these changes make sRACIPE 2.0 a comprehensive and fully integrated suite for RACIPE simulations, while maintaining the ease of use of the R/Bioconductor ecosystem.

## 2 sRACIPE 2.0

**Figure 1:**
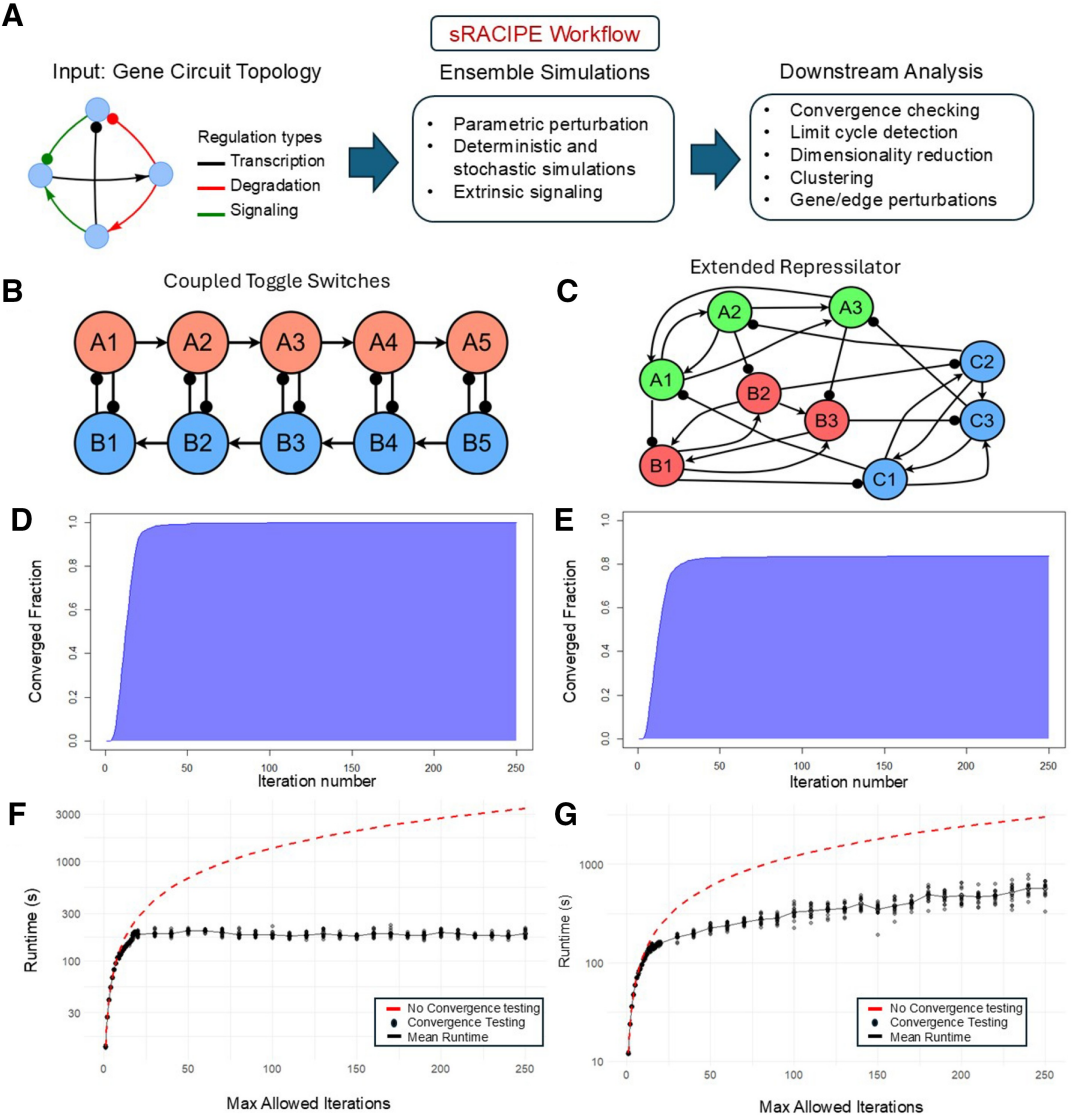
An overview of features in sRACIPE 2.0. (A) sRACIPE workflow. Illustrations of sRACIPE modeling are presented for a coupled toggle switch circuit (panels B, D, F) and an extended repressilator circuit (panels C, E, G). (B, C) Illustration of circuit topologies. Lines and arrowheads represent transcriptional activation, lines and dots represent transcriptional inhibition. (D, E) Cumulative distributions of converged models over simulation iterations. (F, G) Simulation runtime over maximum allowed iterations for cases with or without convergence checking.

sRACIPE 2.0 includes many new features to the sRACIPE package. An illustration of the updated features can be seen in Fig. 1A. Table 1 summarizes the improvements of sRACIPE 2.0 over previous RACIPE packages, and compares it with other simulators that use random parameterizations, such as GeneNetWeaver (Schaffter *et al.* 2011) and HiLoop (Nordick and Hong 2021).

**Table 1:** Feature comparison for various software using random parameterizations, including older implementations of RACIPE.

	sRACIPE 2.0	sRACIPE 1.0	cellcycle	RACIPE	GeneNetWeaver	HiLoop
Purpose	Statistical analysis of GRCs				Inference benchmarking	Motif analysis
Language	R/C++	R/C++	Python/C	C	Java	Python
Stochastic simulation option	✓	✓			✓	
Time-correlated noise	✓					
Degradation regulation	✓		✓			
Oscillation detection	✓		✓			✓
Convergence testing	✓		✓	✓		
Ensemble-based approach	✓	✓	✓	✓		✓

### 2.1 Tools for deterministic analysis

One of the main purposes of the development of sRACIPE 2.0 was to greatly expand tools for deterministic analysis of GRCs. First, we implemented a convergence testing algorithm that allows dynamic runtime, which is achieved by running deterministic simulations on a number of small iterations. During the simulation, after each iteration containing a user-defined number of steps, the algorithm checks if the system has converged using the squared Euclidean distance and ends the simulation early if it has converged. The convergence state of an initial condition and the iterations it took to converge are both recorded in a table in the metadata of the simulation output. Beyond the time efficiency of early stopping, the convergence data can also be used for diagnostic information to help the user determine if they are running the simulation too long (where most models converge before the last iteration) or not long enough (where most models aren’t converging). To enable this diagnostic analysis, we developed a new sracipeConvergeDist() method in sRACIPE 2.0 to visualize convergence data and give statistics on the rate of convergence.

Another important tool for deterministic analysis is the ability to identify the unique steady states for each model in the ensemble. This feature, previously existing in the C and Python versions, has now been implemented in sRACIPE 2.0. Specifically, the sracipeUniqueStates() method collects the unique steady states for each model by running simulations starting from many random initial conditions. To handle the numerical integration error, the algorithm truncates the gene expression values at a user-selected decimal place and uses the indices of the unique rounded expressions to find the unique expressions.

### 2.2 Limit cycle detection analysis

To enable high-throughput detection of oscillatory dynamics across a large ensemble of ODE models, sRACIPE 2.0 now implements a numerical algorithm introduced in a previous study (Katebi *et al.* 2020). For each model that is not yet converged after maximum simulation time, the algorithm continues to run an extended simulation. During the secondary simulation, the algorithm calculates the Euclidean distance between the expression at each time point and at the end of the first simulation. If this scalar distance function is sufficiently periodic, it is counted as a limit cycle and measures the period and expressions along the limit cycle. To prevent multiple counts of the same limit cycle from simulations with different initial conditions, a tolerance was set to account for numerical deviations due to time-step discretization in the ODE integration. This algorithm ensures the rigorous detection of the unique oscillations in a model.

### 2.3 Runtime improvements from convergence checking

We demonstrate the runtime improvements from convergence checking with two synthetic circuits, one being a coupled toggle switch circuit Fig. 1B (Huang *et al.* 2018) for its multistable behavior and the other being an extended repressilator circuit Fig. 1C that exhibits oscillatory dynamics. For each circuit, we first simulated 2000 models for up to 250 maximum iterations and checked how the fraction of converged models increased with respect to the number of simulation iterations. As shown in (Fig. 1D and E), most models converge to steady-states after about 30 iterations for the coupled toggle switch, while 20% of models failed to converge in the extended repressilator due to oscillatory dynamics. Next, we performed runtime measurements on simulations of 250 models with three initial conditions each for both circuits, with varying amounts of the maximum allowable number of iterations. For comparison, we estimated the runtime plot without convergence checking as a line with a slope equal to the average measured runtime for running just one convergence iteration. As shown in Fig. 1F and G, with convergence checking, runtime plateaus or greatly slows down when the simulations are given enough iterations to converge. Thus, the convergence checking method in sRACIPE 2.0 significantly improves simulation efficiency compared to the original test-free approach.

### 2.4 New simulation options

sRACIPE 1.0 only supported transcription factor (TF) regulatory interactions, which influence gene production rates, in GRC models. To allow for more comprehensive GRC modeling, sRACIPE 2.0 now supports three types of gene regulatory interaction types, which include TF interactions, protein degradation (PD) interactions, which influence gene degradation rates, and signaling interactions, which regulate production at a faster rate than TF interactions. TF, PD, or signaling regulations can greatly impact on the dynamical behavior of a GRC model, as demonstrated by five synthetic circuits in Figs 1 and 2, available as supplementary data at *Bioinformatics* online and the yeast cell cycle circuit from (Katebi *et al.* 2020) in Fig. 3, available as supplementary data at *Bioinformatics* online.

While the original sRACIPE had options for stochastic simulations using SDEs, it is limited to modeling Gaussian white noise. Several recent works have studied time-correlated stochastic processes such as the Ornstein–Uhlenbeck (OU) process in GRC dynamics (Thattai and van Oudenaarden 2001, Paulsson 2004, Sharma and Dutta 2017). Therefore, the new sRACIPE includes a modeling option for OU noise, enabling exploration of how uncorrelated versus temporally correlated fluctuations influence GRC dynamics. A case study of how these noise types impact dynamics can be found in Fig. 4, available as supplementary data at *Bioinformatics* online and Table 1, available as supplementary data at *Bioinformatics* online.

For modeling gene perturbation of GRCs, in addition to the knockdown and knockout simulations in sRACIPE 1.0, sRACIPE 2.0 introduces several new tools for this analysis. The first is the use of time-varying production and degradation parameters for selected genes. Specifically, the user can define the production rate *g* or degradation rate *k* as $g=g_{0}x(t)$ or $k=k_{0}y(t)$ for selected genes. In this case, $g_{0}$ and $k_{0}$ are randomly sampled as normal in RACIPE for each model, while x(t) and y(t) are the same across the ensemble. The user defines x(t) by giving a vector $T=(0,t_{1},\ldots ,t_{n})$, where $t_{n}$ is the end of the simulation, and for each altered parameter a vector $X=(x_{0},x_{1},\ldots ,x_{n})$ of output values. In the simulation, the value of x(t) is calculated by linear interpolation using T and X. This time dependence can be used to emulate dynamic extrinsic signaling inputs or environmental changes, shedding light on transient responses that static models may miss. The second new option for perturbation analysis in sRACIPE 2.0 is the ability to force selected genes to be held constant during the simulation (referred to as gene clamping). The user can either clamp the gene at a different value for each model or have the value constant over the entire ensemble, which mimics experimental perturbations like gene knockout, knockdown and overexpression. The perturbation analysis is illustrated using a toggle switch in Fig. 5, available as supplementary data at *Bioinformatics* online.

## 3 Design and implementation

sRACIPE 2.0 is a major update to the foundations of our previous sRACIPE R/Bioconductor package (Kohar and Lu 2018). The package workflow revolves around loading the input circuit in R and performing complex computational tasks in C++, including the integration of ODEs and SDEs, parameter selection, and limit cycle detection (Fig. 6, available as supplementary data at *Bioinformatics* online). An updated version of RacipeSE, which extends the common S4 SummarizedExperiment class (Huber *et al.* 2015), was used to store simulation data, including raw expressions, model parameters, network topology, limit cycle information, and convergence data.

## 4 Conclusion and discussion

sRACIPE 2.0 is a user-friendly R package that only requires the topology of a GRC as input. The updated toolkit enables the integration of diverse gene interaction types, detection of both stable steady states and limit cycles, advanced diagnostic tools for deterministic simulations, and methods for simulating time-correlated intrinsic noise and extrinsic signaling. These new features make sRACIPE 2.0 a powerful unified toolkit for ensemble-based systems biology modeling of gene regulatory circuits. There are also a few areas to extend the abilities of sRACIPE in the future. One option is introducing other types of gene interactions, such as microRNA-based post-transcriptional regulation. Furthermore, it is challenging to integrate stiff ODEs, which can arise in large, dense GRCs. To alleviate this issue, we could implement more integration options to improve accuracy. Lastly, we intend to integrate the updated sRACIPE 2.0 with a web app named Gene Circuit Explorer (GeneEx) for analyzing GRCs from a web browser (Kohar *et al.* 2021).

## Supplementary Material

btag019_Supplementary_Data

## Data Availability

All data underlying the algorithm development, modeling scripts and simulated data are available in Zenodo at https://doi.org/10.5281/zenodo.18202342.
