# Supplementary material for: sRACIPE 2.0: a systems biology circuit modeling toolkit for random circuit perturbation
Source: Bioinformatics. 2026 Jan 19;42(2):btag019. doi: 10.1093/bioinformatics/btag019 (PMC12866642; doi:10.1093/bioinformatics/btag019)
Supplement: btag019_Supplementary_Data [file btag019_supplementary_data.zip › 22-Jan-2026_110722_sRACIPE_2_0_SI.pdf]

sRACIPE 2.0: a system biology circuit modeling toolkit for random circuit perturbation

Supplemental Information

Aidan Tillman, Daniel Ramirez, and Mingyang Lu

SI Text

Mathematical models in sRACIPE 2.0

The suite for Random Circuit Perturbation (sRACIPE) 2.0 package is a user-friendly tool to comprehensively study gene regulatory networks (GRCs) by randomly sampling kinetic parameters to construct an ensemble of Differential Equation (DE) models for the circuit. Statistical analysis of simulations on these models elucidates the stability and basin of attractions of the gene expression state for these models. For details on the exact structure, use cases, and classes the package uses, consult the package vignettes.

Mathematical Model

The Differential equations used by the package are a mathematical model of the degradation and production rates of expression for a single gene, or node, in a given GRC. The expression for an isolated node with no inward interactions, A, the differential equation is

$$\frac{dA}{dt} = g_A - k_A A, \tag{S1}$$

with g_A and k_A are the basal production and degradation rates of gene A, respectively. The interaction types currently modeled by sRACIPE involve regulating these production and degradation terms. These interactions are modeled using shifted Hill functions (Huang et al. (2017)). For a gene A being regulated by a gene B, these are defined as

$$H_s(B; T_{AB}, n_{AB}, \lambda_{AB}) = \lambda_{AB} + (1 - \lambda_{AB}) \frac{1}{1 + (\frac{B}{T_{AB}})^{n_{AB}}}, \tag{S2}$$

with T_{AB}, n_{AB}, λ_{AB} as the threshold constant, the Hill coefficient, and the fold change of the regulation respectively. The Hill function is inhibitory if $0 < \lambda_{AB} < 1$, denoted by H^-. For $\lambda_{AB} > 1$, it is excitatory, denoted by H^+. Transcription factors regulations are modeled by multiplying the production term in equation S1:

$$\frac{dA}{dt} = H_S(B)g_A - k_A A \tag{S3}$$

Signaling interactions also regulate the production term, but are typically faster than transcription factor or protein degradation interactions, so if there are only inward signaling interactions on gene A, then both the reaction terms are multiplied by a rate constant η to account for this speed difference (Katebi et al. (2020)):

$$\frac{dA}{dt} = \eta(H_S(B)g_A - k_A A) \tag{S4}$$

In sRACIPE, η is a simulation hyperparameter held constant across the ensemble. It is denoted "signalRate" in the sracipeSimulate() function and its default value is 10.0. Lastly, protein degradation interactions involve influencing the degradation term of equation S1:

$$\frac{dA}{dt} = g_A - H_S(B)k_A A \tag{S5}$$

sRACIPE uses multiplicative logic in the case of multiple inward interactions. Thus if gene A has n_1 transcription factor interactions (X_i), n_2 signaling interactions (Y_j), and n_3 protein degradation interactions (Z_l), then the resultant differential equation is

$$\frac{dA}{dt} = \left(\prod_{i=1}^{n_1} H_s(X_i)\right)\left(\prod_{j=1}^{n_2} H_s(Y_j)\right) g_A - \left(\prod_{l=1}^{n_3} H_s(Z_l)\right) k_A A. \tag{S6}$$

Lastly, the degradation and production rates k and g are represented in terms of the maximal degradation and production rates K and G, where are the rates when activators are abundant and inhibitors are scarce. When gene A has n_1 activators of production (X_i) (either transcription factor or signaling interaction) and n_2 activators of protein degradation (Y_i), then $g_A = \frac{G_A}{\prod_{i=1}^{n_1} \lambda_{AX_i}}$ and $k_A = \frac{K_A}{\prod_{i=1}^{n_2} \lambda_{AY_i}}$. Thus, for each gene, there are two kinetic parameters G and K; for each gene interaction, there are three kinetic parameters T, n, and λ. Each model in the ensemble generated by sRACIPE is defined by the uniform random sampling of these parameters. The package then integrates these models using either the Euler, 4th order Runge-Kutta or adaptive-step Dormand-Prince integration methods to find the possible steady states of the circuit.

Stochastic Models

Optionally, the package has two options to simulate the GRC using stochastic differential equations (SDEs) as well. The first is the simple addition of white noise by adding a Wiener process W_t with variance σ^2 to the reaction terms (Kohar and Lu (2018)):

$$\frac{dA}{dt} = \left(\prod_{i=1}^{n_1} H_s(X_i)\right)\left(\prod_{j=1}^{n_2} H_s(Y_j)\right) g_A - \left(\prod_{l=1}^{n_3} H_s(Z_l)\right) k_A A + \sigma dW_t \tag{S7}$$

The second option is the use of time-correlated noise with an Ornstein-Uhlenbeck process:

$$\frac{dA}{dt} = \left(\prod_{i=1}^{n_1} H_s(X_i)\right)\left(\prod_{j=1}^{n_2} H_s(Y_j)\right) g_A - \left(\prod_{l=1}^{n_3} H_s(Z_l)\right) k_A A + U(t), \tag{S8}$$

where $U(t)$ is defined recursively as

$$U(t+h) = U(t)\cdot e^{-\frac{h}{\tau}} + \sigma\sqrt{1-e^{-\frac{2h}{\tau}}}\cdot W_t, \tag{S9}$$

where, as before, W_t is a Wiener process with variance σ^2, h is the integration step size, and $\tau > 0$ is the time correlation of the noise which modulates the rate of mean-reversion. h, τ and σ^2 are adjustable parameters in the simulation.

Estimating transition rate from stochastic simulations

We demonstrated the effects of different noise models by simulating a toggle switch model with fixed symmetric parameters and evaluating transition rates between states under each noise model via mean first passage time (MFPT). The parameters of the model used were as follows: $G = 70$, $k = 0.2$, $X_0 = 5$, $n = 3$, $\lambda = 80$; where G denotes production rate, k denotes degradation rate, X_0 is threshold level, n is Hill coefficient, and λ is fold change. To estimate t_{MFPT}, we simulated stochastic trajectories under both OU and white noise for a total duration of $t = 30000$ and recorded snapshots every 2 unit time. Because the model was bistable and symmetric, we measured the distance between states as $q = A - B$ for every time point, with $q = 0$ representing the boundary between basins. The time indices where the trajectory crossed $t = 0$ were selected, and the first 200 unit time after each crossing was discarded before re-arming, to prevent counting re-crossings. The average time from re-arming to the next crossing was taken as the MFPT. Finally, the transition rate is computed as in (Hänggi et al. (1990)):

$$k = \frac{1}{2t_{MFPT}}. \tag{S10}$$

SI Figures

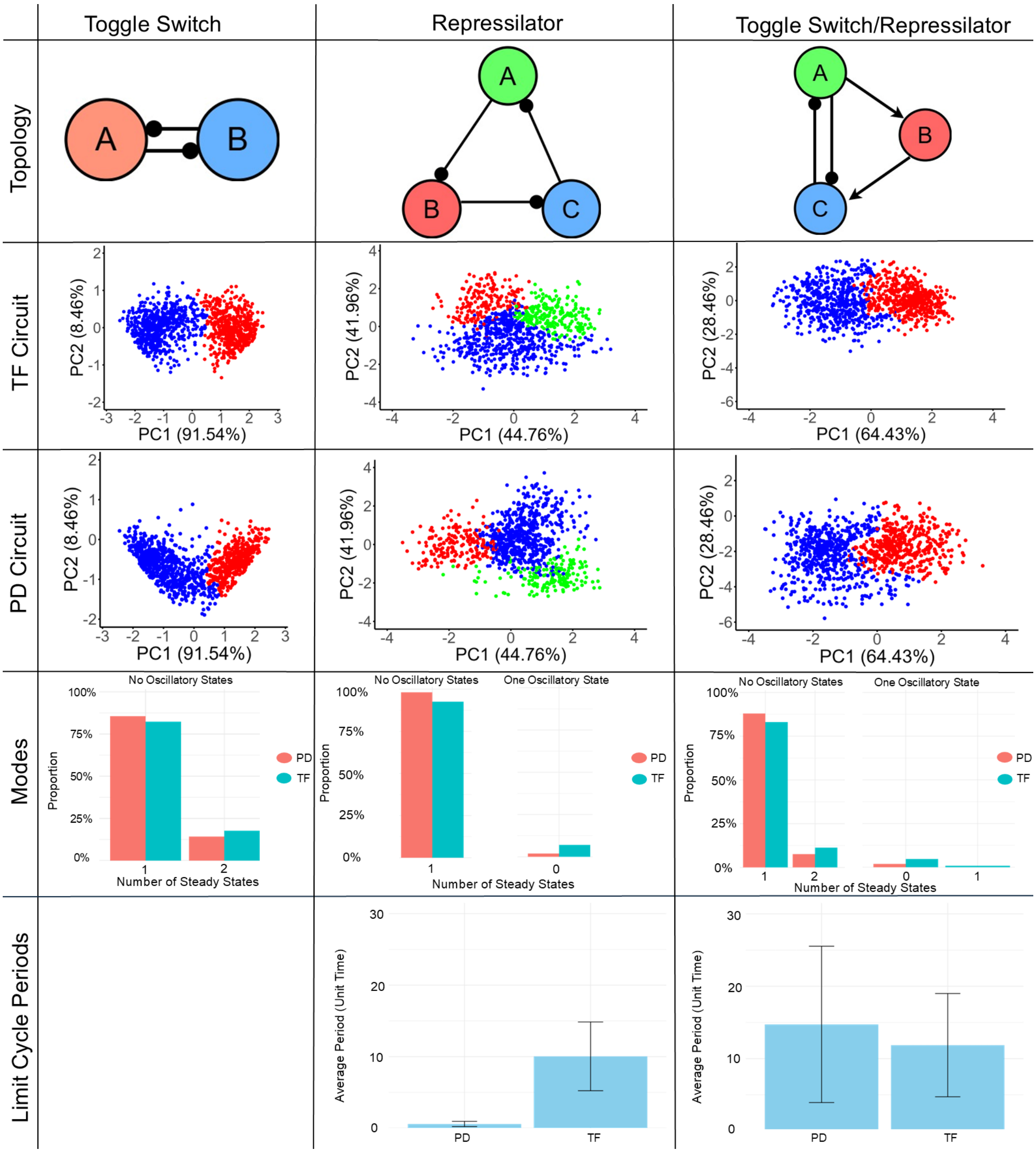
Toggle Switch
Repressilator
Toggle Switch/Repressilator
Topology
TF Circuit
PD Circuit
Modes
Limit Cycle Periods
PC1 (91.54%)
PC2 (8.46%)
PC1 (44.76%)
PC2 (41.96%)
PC1 (64.43%)
PC2 (28.46%)
No Oscillatory States
One Oscillatory State
Number of Steady States
Proportion
Average Period (Unit Time)
PD
TF

Figure S1: Simulation results for three small synthetic GRC topologies under transcription factor (TF) and protein degradation (PD) regulation regimes. The Toggle Switch (leftmost column) was chosen for exhibiting bistability, the Repressilator (middle column) was chosen for exhibiting oscillations, and the Toggle Switch/Repressilator (rightmost column) was chosen for exhibiting both bistability and oscillations. 1000 models were simulated with 100 initial conditions each for each ensemble. The first row has circuit diagrams for each topology. The second and third rows show Hierarchical clustering (using Euclidean distance and Ward's linkage method) results for simulated stable steady states for the TF and PD circuit for each topology. To emphasize geometric differences between clusters, both circuit results were projected along the PCA components of the TF circuit. The fourth row is a comparison of dynamical modes found across each ensemble for each topology. The last row compares average period of oscillation for each ensemble with the standard deviation for error bars. The Repressilator exhibits faster oscillations in the PD circuit, while the Toggle Switch/Repressilator exhibits similar averages but more variance in the PD circuit. PD interactions directly affect the degradation kinetics, with PD inhibition acting by activating degradation. Hence, the Repressilator with only inhibition interactions has faster degradation kinetics in the PD circuit, decreasing the time scale, and the Toggle Switch/Repressilator with an equal amount of activation and inhibition interactions will have a similar average time scale in the TF and PD circuits, but with higher variance due to time scale depending on sampling of the fold change λ.

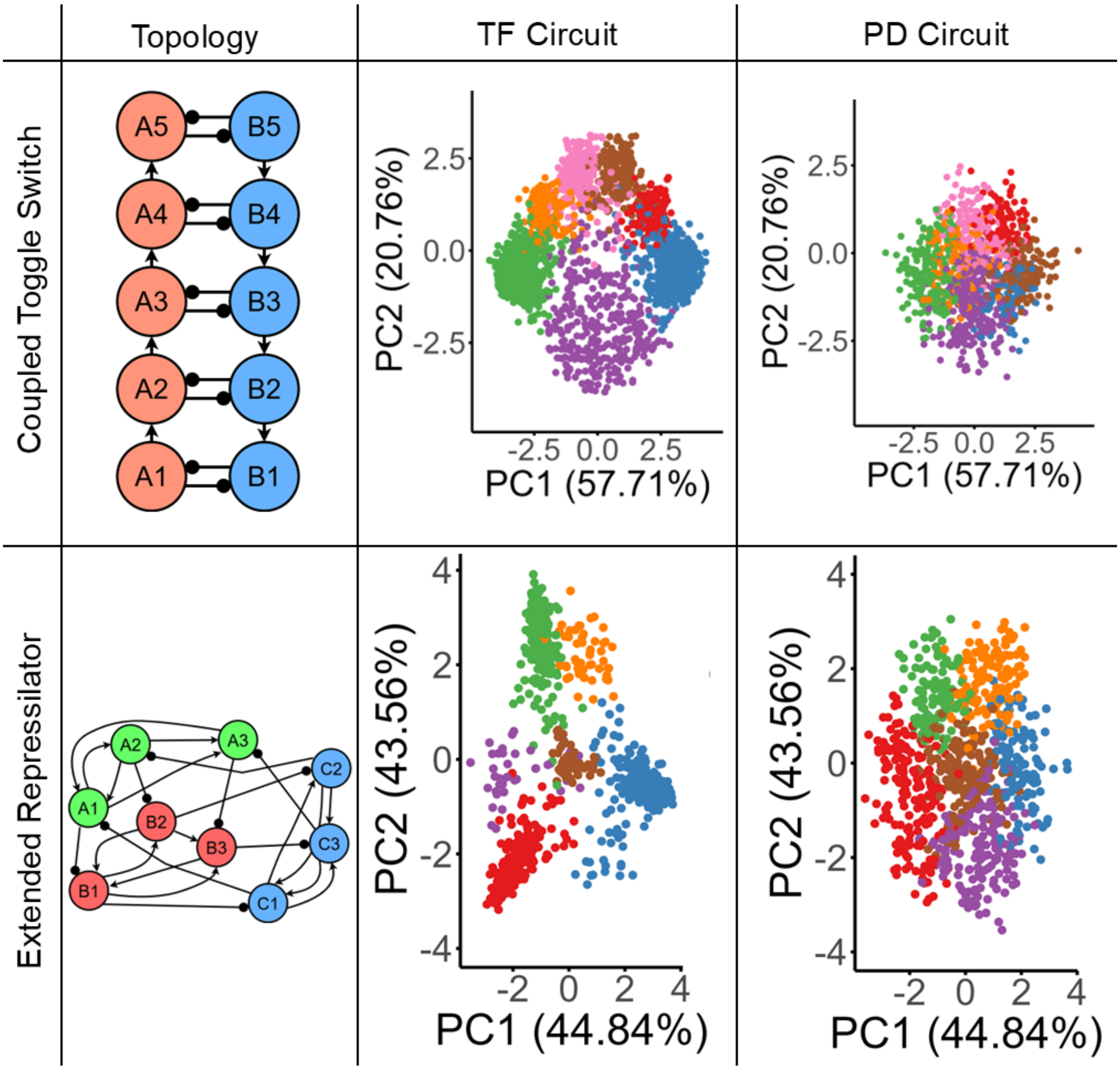

Figure S2: Simulation results for the Coupled Toggle Switch and Extended Repressilator topologies under transcription factor (TF) and protein interaction (PD) regulation regimes. The fold change λ is sampled from 1 to 100 for TF interactions and 1 to 10 for PD interactions. The first column has circuit diagrams, and the second and third columns show PCA and hierarchical clustering using the Euclidean distance and Ward's method for linkage were performed, with both ensembles projected along the PCA components for the TF data to emphasize the geometric differences of the data. Noisy clusters were observed for the simulations of the circuit with PD regulations due to the PCA projection. Scripts for these simulations and analysis can be found in `https://github.com/dan-ramirez-23/sRACIPE-Demos`.

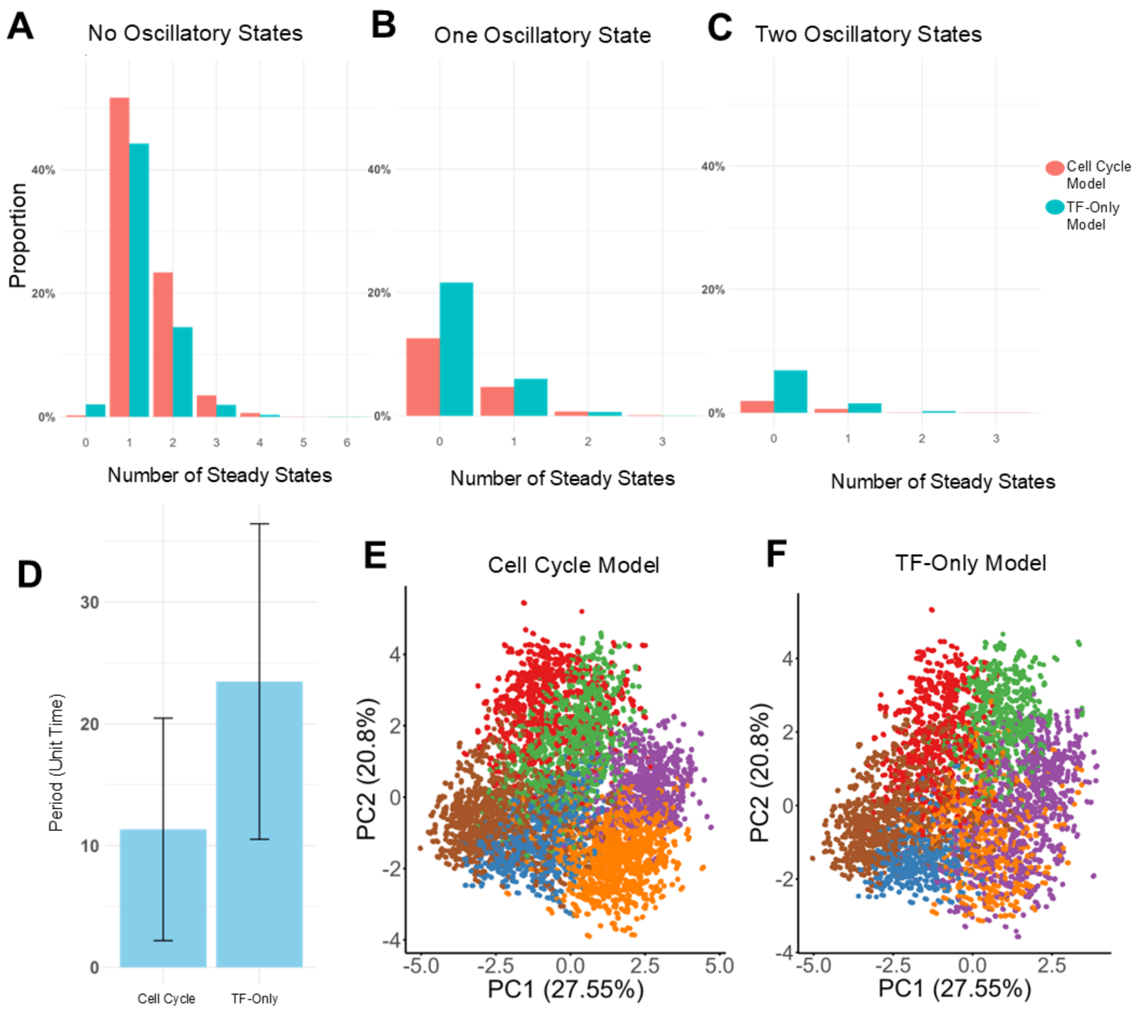

Figure S3: A comparison of two different regulatory regimes for the cell cycle from (Katebi et al. (2020)), a complete cell cycle model containing TF, PD, and signaling interactions, and a model where every link is treated as a TF regulation. 10,000 models were generated per ensemble with 100 initial conditions each. The vignette for these simulations can be found in `https://github.com/dan-ramirez-23/sRACIPE-Demos`. (**A,B,C**) A comparison of dynamical modes found across each ensemble for each model. For each model, we specified sRACIPE to only detect no more than two oscillatory states. Less than 5% of models failed to converge due to short runtime. (**D**) Average periods of oscillations in each model, with standard deviation as error bars. (**E,F**) Hierarchical clustering using Euclidean distance and Ward's linkage method on PCA projections of the steady states for each model, with the TF-only model projected along the PCA components for the diverse model to get a clear comparison of cluster shapes. The six gene expression clusters were associated with six cellular states during cell cycle progression, as identified in (Katebi et al. (2020)). Color in the PCA projections corresponds to different clusters.

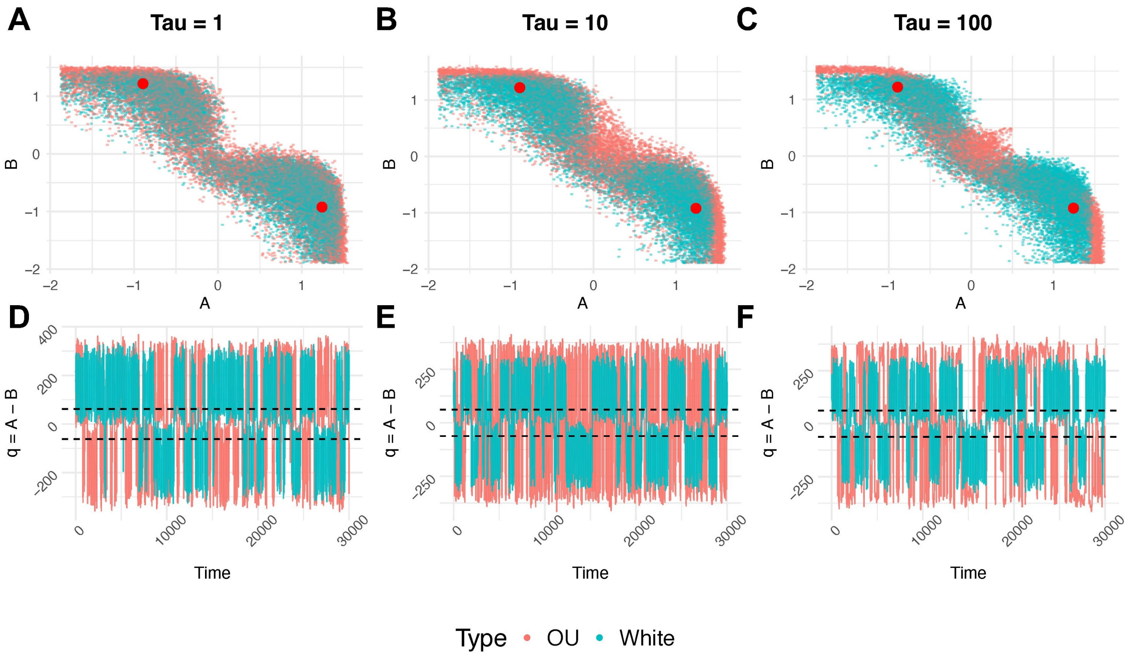

Figure S4: Properties of state transitions in a toggle switch with different types of gene expression noise. A-C) Scatterplots of the time trajectories for stochastic simulations of a single symmetric toggle switch model using white noise (blue) and OU noise (red), with normalized values of nodes A and B on the x and y axes. Large red points indicate the location of the two stable steady states. Panels show data for tau of 1, 10, and 100, respectively. **D-F)** Time series value of q (defined as $A - B$) for the model shown in the panels above. Blue time course corresponds to white noise, and red corresponds to OU noise. Dashed lines indicate the middle 30% of values of q, i.e. 15% of the distance from 0 toward each stable steady state.

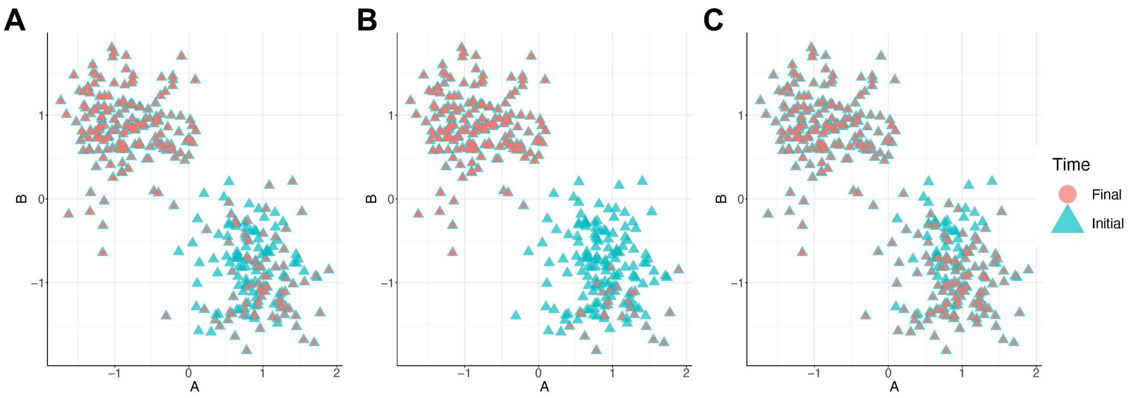

Figure S5: Perturbation simulations to drive state transitions in a toggle switch circuit. A) Scatterplot of normalized stable steady states before and after simulating the same models with the value of node B clamped at the value of the high steady state until convergence, followed by a relaxation phase with clamps removed for models to converge again. Blue circles mark initial stable steady states; red triangles mark the stable steady states after transient perturbation. **B)** Scatterplot of stable steady states of the circuit before and after signaling induction with paired initializations, i.e. two instances of the model with one initial condition set to each stable steady state. During the simulation, production of gene B was modified linearly from 1-fold up to 10-fold and back between $t = 1$ and $t = 50$, followed by a relaxation phase from $t = 50$ to $t = 200$. **C)** Scatterplot of stable steady states of the circuit before and after the signaling induction identical to panel **(B)** but with a lower maximal fold-change of 2 rather than 10. All simulations in this figure are from deterministic simulations, but transition efficacy could be modulated by adjusting the timescale, strength of signals (or number of clamped genes), or noise level during simulation.

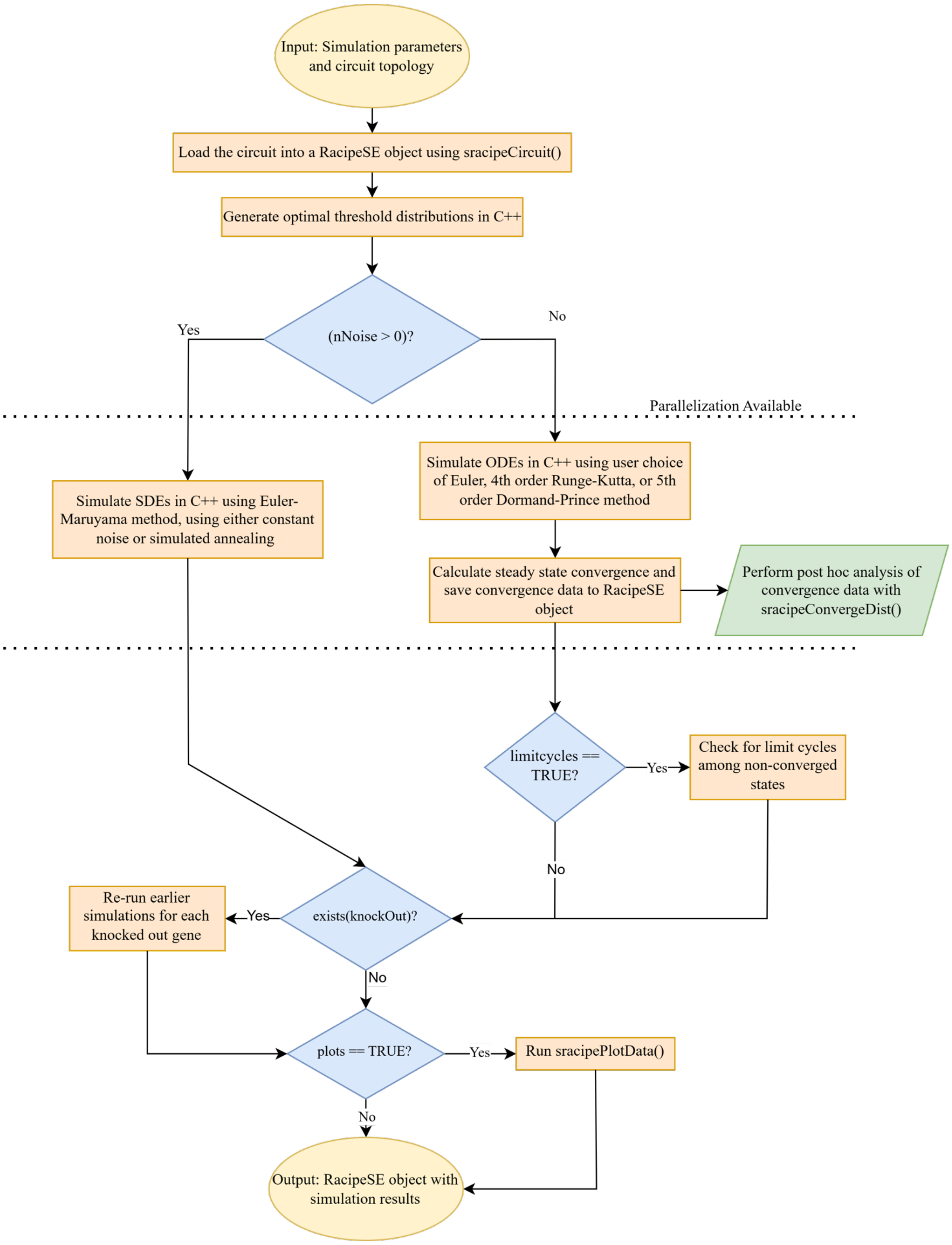

Figure S6: Workflow diagram for sracipeSimulate, the main function for sRACIPE 2.0, based on user-provided inputs. For enhanced computational efficiency, parallelization is implemented using the doFuture (Bengtsson (2021)) and doRNG (Gaujoux (2024)) packages. Parallelization is also supported with the sracipeCombineRacipeSE() function. R and C++ are connected using the Rcpp package (Eddelbuettel and Francois (2011)). Simulation results are stored in the RacipeSE class, which is an extension of the common S4 class SummarizedExperiment.

SI Table

Noise Type	Amplitude	Tau (OU)	Transition Rate	Transition Rate SE	MFPT	Median FPT
White	0.15	–	$1.13*10^{-3}$	$2.09*10^{-5}$	443.0	295.0
		–	$7.40*10^{-4}$	$2.29*10^{-5}$	676.0	448.0
		–	$1.11*10^{-3}$	$2.29*10^{-5}$	448.0	333.0
OU		1	$2.20*10^{-3}$	$3.11*10^{-5}$	227.0	170.0
		10	$5.85*10^{-3}$	$6.07*10^{-5}$	85.5	60.5
		100	$2.44*10^{-3}$	$3.34*10^{-5}$	226.0	154.0

Table S1: Summary of state transition properties for a toggle switch circuit under different noise types. The results are related to the simulated trajectories shown in **Fig. S4**. Six trajectories were simulated for 30000 unit time with snapshots taken every 2 unit time to estimate the transition rate via (Hänggi et al. (1990)) and MFTP. The column named "Transition Rate SE" shows the standard deviation of the transition rate.

References

H. Bengtsson. A unifying framework for parallel and distributed processing in r using futures. *The R Journal*, 13(2):208–227, 2021. doi: 10.32614/RJ-2021-048.

D. Eddelbuettel and R. Francois. Rcpp: Seamless r and c++ integration. *Journal of Statistical Software*, 40(8):1–18, 2011. doi: 10.18637/jss.v040.i08.

R. Gaujoux. *doRNG: Generic Reproducible Parallel Backend for 'foreach' Loops*, 2024. R package version 1.8.3.

P. Hänggi, P. Talkner, and M. Borkovec. Reaction-rate theory: Fifty years after Kramers. *Reviews of Modern Physics*, 62(2):251–341, Apr. 1990. ISSN 0034-6861, 1539-0756. doi: 10.1103/RevModPhys.62.251.

B. Huang, M. Lu, D. Jia, E. Ben-Jacob, H. Levine, and J. N. Onuchic. Interrogating the topological robustness of gene regulatory circuits by randomization. *PLOS Computational Biology*, 13(3): e1005456, 2017. doi: 10.1371/journal.pcbi.1005456. URL `https://app.dimensions.ai/details/publication/pub.1084271199`.

A. Katebi, V. Kohar, and M. Lu. Random Parametric Perturbations of Gene Regulatory Circuit Uncover State Transitions in Cell Cycle. *iScience*, 23(6):101150, 2020. ISSN 2589-0042. doi: https://doi.org/10.1016/j.isci.2020.101150. URL `https://www.sciencedirect.com/science/article/pii/S2589004220303357`.

V. Kohar and M. Lu. Role of noise and parametric variation in the dynamics of gene regulatory circuits. *npj Systems Biology and Applications*, 4(1):40, 2018. ISSN 2056-7189. doi: 10.1038/s41540-018-0076-x. URL `https://doi.org/10.1038/s41540-018-0076-x`.
